# Activation of NF-κB signaling by the dense granule protein GRA15 of a newly isolated type 1 *Toxoplasma gondii* strain

**DOI:** 10.1186/s13071-022-05429-x

**Published:** 2022-09-29

**Authors:** Guanghao Guo, Jianmin Cui, Lindong Song, Lvqing Tang, Sijie Fan, Bang Shen, Rui Fang, Min Hu, Junlong Zhao, Yanqin Zhou

**Affiliations:** 1 Key Laboratory Preventive Veterinary of Hubei Province, College of Veterinary Medicine, Huazhong Agricultural University, Wuhan, 430070 Hubei People’s Republic of China; 2 State Key Laboratory of Agricultural Microbiology, Huazhong Agricultural University, Wuhan, 430070 Hubei People’s Republic of China

**Keywords:** *Toxoplasma gondii*, Toxoplasmosis, NF-κB, GRA15

## Abstract

**Background:**

It has been reported that the NF-κB pathway, an important component of host defense system against pathogens infections, can be differentially modulated by different *Toxoplasma gondii* strains, depending on the polymorphism of the GRA15 protein. The recently isolated *Toxoplasma* strain T.gHB1 is a type 1 (ToxoDB#10) strain but shows different virulence determination mechanisms compared to the classic type 1 strains like RH (ToxoDB#10). Therefore, it is worth investigating whether the T.gHB1 strain (ToxoDB#10) affects the host NF-κB signaling pathway.

**Methods:**

The effects of T.gHB1 (ToxoDB#10) on host NF-κB pathway were investigated in HEK293T cells. The GRA15 gene product was analyzed by bioinformatics, and its effect on NF-κB activation was examined by Western blotting and nuclear translocation of p65. Different truncations of T.gHB1 GRA15 were constructed to map the critical domains for NF-κB activation.

**Results:**

We demonstrated that the NF-κB pathway signaling pathway could be activated by the newly identified type 1 T.gHB1 strain (ToxoDB#10) of *Toxoplasma*, while the classic type 1 strain RH (ToxoDB#10) did not. T.gHB1 GRA15 possesses only one transmembrane region with an extended C terminal region, which is distinct from that of classic type 1 (ToxoDB#10) and type 2 (ToxoDB#1) strains. T.gHB1 GRA15 could clearly induce IκBα phosphorylation and p65 nuclear translocation. Dual luciferase assays in HEK293T cells revealed a requirement for 194–518 aa of T.gHB1 GRA15 to effectively activate NF-κB.

**Conclusions:**

The overall results indicated that the newly isolated type 1 isolate T.gHB1 (ToxoDB#10) had a unique GRA15, which could activate the host NF-κB signaling through inducing IκBα phosphorylation and p65 nuclear translocation. These results provide new insights for our understanding of the interaction between *Toxoplasma* parasites and its hosts.

**Graphical Abstract:**

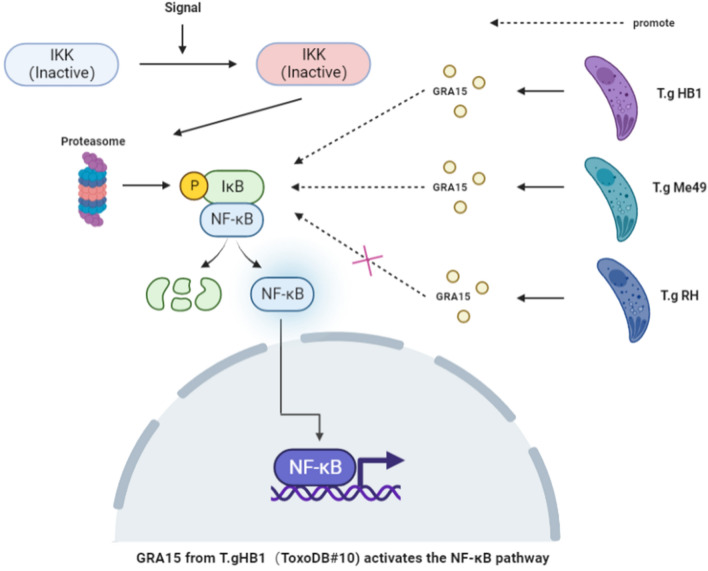

## Background

*Toxoplasma gondii* is a ubiquitous obligate intracellular parasite that infects a wide range of warm-blooded animals and humans [1]. Despite a large number of intermediate hosts, only domestic cats and other feline animals can serve as its definitive hosts [2]. Human infection is generally acquired by ingesting undercooked meat containing tissue cysts or water, fruits or vegetables contaminated with oocysts [3]. It is established that 30% of the world’s population was seropositive for toxoplasmosis. Infection by *T. gondii* is usually benign and asymptomatic in healthy individuals. While severe outcomes and even death may occur when *Toxoplasma* infection happens in those with compromised immunity or organ transplanted recipients [4].

Despite its high prevalence in the world, only one specie for the genus of *Toxoplasma* has been defined [5]. Thus far, > 200 *Toxoplasma* genotypes have been recorded in the *T. gondii* database (http://toxodb.org/toxo). Genotyping analysis showed a rich genetic diversity within worldwide strains, suggesting the distribution of *T. gondii* genotypes may vary greatly with geographical location [6]. The population structure of *Toxoplasma gondii* was originally thought to consist of three main clonal lineages (types 1, 2 and 3) [7]. In Europe and North America, types 2 and 3 are the dominant strains (as well as type 12 in North America). In South and Central America, there are many atypical (non-clonal) strains with no clear genotype dominant [8]. When assessed in mice, the virulence of different *T. gondii* strains differs significantly. Type 1 strains cause 100% mortality at infection dose as low as one parasite per mouse. Type 2 and 3 strains cause moderate or no mortality depending on doses [9]. A recent study showed that isolates from North America, Europe, North Africa and Asia were not lethal to mice at low infective doses, while isolates from South America were mostly lethal to mice [10]. In China, *Toxoplasma* isolates displayed limited genetic diversity but showed diverse pathogenicity to mice. Genotyping analysis showed that Chinese 1 lineage (ToxoDB#9) ranks as the most prevalent isolate among all known strains [11–13].

*Toxoplasma* invasion, growth and development inside host cells involved complex interactions between host and parasites [14–16]. Previous studies have shown that different strains of *Toxoplasma* parasites can secrete different effectors into host cells and participate in modulating different host signaling pathways [17, 18]. Other studies revealed that this diversification was partly caused by polymorphic parasitic effects. For example, in a recent report, Zhang et al*.* described a novel *Toxoplasma* isolate T.gHB1 (ToxoDB#10), which is a type 1 strain determined by RFLP genotyping [19]. However, it possesses different virulence determinant mechanisms as a combined deletion of the parasite’s GRA7 plus ROP17 or GRA7 plus ROP18 genes only slightly attenuated parasite virulence in mice. Deleting the same sets of genes in the classic type 1 strain RH (ToxoDB#10) drastically reduced parasite virulence in mice [20, 21]. These data suggest that T.gHB1 (ToxoDB#10) possesses polymorphic properties compared to other typical type 1 strains.

The NF-κB pathway plays an important role in host innate and adaptive immune systems. Earlier studies have suggested that many pathogens have evolved numerous strategies to modulate this host NF-κB signaling for immune evasion or maintaining a safe shelter [22, 23]. For *Toxoplasma* parasites, type 2 but not the type 1 and type 3 parasites can activate host NF-κB pathway, which is determined by the parasite’s polymorphic dense granule protein called GRA15. Mice infected with a type 2 GRA15KO strain had a significantly higher parasite burden than mice infected with a type 2 strain. Type 2 GRA15s induce NF-κB activation, increasing cell migration, thereby recruiting new cells to infect. Furthermore, NF-κB-mediated inflammation leads to tissue damage, allowing *Toxoplasma* to cross tissue barriers [24]. The potential of T.gHB1 to modulate host NF-κB signaling cascade has not been investigated thus far. In the current study, we demonstrated that T.gHB1 imposed a positive effect on activating host NF-κB pathway. Our results also showed that T.gHB1 GRA15 contains one transmembrane region and an extended C-terminal region (518–602 aa) compared to the type 2 strains. Furthermore, our results showed that GRA15 alone efficiently activated NF-κB and resulted in IκBα phosphorylation and p65 nuclear translocation. This process required 194–518 aa of GRA15. In contrast, our studies showed an inhibitory effect of the typical type 1 strain RHΔHX on NF-κB. Taken altogether, our results demonstrate the activation of NF-κB by the recently discovered T.gHB1 type 1 strain and suggest the divergence in NF-κB signaling modulation by different type 1 *Toxoplasma* strains.

## Methods

### Parasites and cells

*Toxoplasma* parasites including T.gHB1 (ToxoDB#10) were maintained in vitro by serial passaging on monolayers of human foreskin fibroblast HFF cells in a 37 °C incubator with 5% CO_2_. RHΔHX (ToxoDB#10) and ME49 (ToxoDB#1) strains were used as representative type 1 strain and type 2 strain, respectively. HFF and human kidney epithelial HEK293T cells were cultured in Dulbecco’s Modified Eagle Medium (DMEM) supplemented with 10% FBS (Gibco, Australia), 2 mM L-glutamine, 100 µg/ml penicillin and 100 U/ml streptomycin. Cells and parasites were routinely checked for *mycoplasma* contamination and found to be negative.

### Plasmid construction

Primers were designed by using Primer Premier 5.0, and the full-length coding sequences for GRA15 from RHΔHX, ME49 and T.gHB1 strains were amplified from tachyzoites cDNA by RT-PCR. The predicted ORF of the respective strains was ligated into the pCMV-tag2B expression plasmid with N terminally tagged with FLAG, and the recombinant plasmid was named pCMV-GRA15 (HB1), pCMV-GRA15 (Me49) and pCMV-GRA15 (RH). To further identify the potential domains of GRA15 required for host signaling modulation, the pCMV-GRA15 (HB1) plasmid was used as template for PCR amplification to generate the desired truncated protein sequences. The resulting sequences were ligated into pCMV-tag2B plasmid as described above to generate the pCMV-GRA15-1, pCMV-GRA15-2, pCMV-GRA15-3, pCMV-GRA15-4 and pCMV-GRA15-5 plasmids as illustrated in Fig. 5a. The primers used in this study are listed in Table 1. All plasmids used in this study were verified by sequencing and prepared using endotoxin free plasmid kit according to the manufacturer’s recommendations (Omega, USA).

**Table 1:** Primer sequences of GRA15 genes

Plasmids	Primers
GRA15(HB1)	F: AGCCCGGGCGGATCCATGGTGACAACAACCACGC R: GGTATCGATAAGCTTTCATGGAGTTACCGCTG
GRA15(Me49)	F: AGCCCGGGCGGATCCATGGTGACAACAACCACGC R: GGTATCGATAAGCTTTCATGGAGTTACCGCTG
GRA15(RH)	F: AGCCCGGGCGGATCCATGGTGACAACAACCACCC R: GGTATCGATAAGCTTTCAACGATGTCCCCTCA
pCMV-GRA15-1	F: ACCCCACATCCTCACAACACACC R: GGTATCGATAAGCTTTACTGCATACGCTCCTAGC
pCMV-GRA15-2	F: AGCCCGGGCGGATCCATGGTGACAACAACCACGC R: GGTATCGATAAGCTTTGAGTCCTTTCTTIGTGC
pCMV-GRA15-3	F: AGCCCGGGCGGATCCATGGTGACAACAACCACGC R: GGTATCGATAAGCTTCTCAGTTGGTACAGAGTAG
pCMV-GRA15-4	F: AGCCCGGGCGGATCCATGGGTAGTGATAGCCGGAACC R: GGTATCGATAAGCTTCTCAGTTGGTACAGAGTAG
pCMV-GRA15-5	F: AGCCCGGGCGGATCCATGGGTAGTGATAGCCGGAACC R: GGTATCGATAAGCTTTCATGGAGTTACCGCTG

### Dual luciferase assay

To investigate NF-κB activation, the dual luciferase approach was adopted to detect the NF-κB promoter activity. Briefly, HEK293T cells were grown in 24-well cell culture plates at 60–80% confluency. For parasite treatment, pNF-κB-Luc plasmids containing firefly luciferase gene under the control of NF-κB promoter and pRL-TK, which acts as an internal reference, were co-transfected into HEK293T cells by Lipofectamine 2000 (Invitrogen, USA). Six hours later, newly egressed tachyzoites were harvested, counted and added into the HEK293T cells as indicated; 24-h post parasites infection, cells were collected and lysed, and luciferase activities were measured as recommended (Promega, USA). To test the influence of RHΔHX on NF-κB signaling, HEK293T cells were infected with RHΔHX parasites; ME49 was used as a positive control. Eighteen hours post-infection, human recombinant TNF-α was added at a final concentration of 20 ng/ml and lasted for 6 h. Then, luciferase activities were detected as described above. HEK293T cells were co-transfected with NF-κB and pRL-TK luciferase reporter plasmid along with each of the GRA15-containing plasmids, and after 24 h, the luciferase activity was measured in cell lysates and all repeated three times independently.

### Parasite invasion assay

To eliminate the possibility that the diversification was caused by differences in parasite invasion as each strain may have different invasion ability in cells currently in use, parasite invasion rates were evaluated. To do so, HEK293T cells grown on coverslips (coverslips were pre-coated with poly-l-lysine to enhance cell adhesion and avoid washing away in the following process) were infected with each parasite strain at MOIs of 2, 3, 6 and 9. One hour later, the extracellular parasites were washed away and continued to grow to 18 h. Then, the coverslips were fixed and detected by differential staining methods. The average parasite invasion rate was calculated by this formula: parasite invasion rate = (total parasite parasitophorous vacuole numbers—extracellular parasite numbers)/total host cell numbers [25].

### Immunofluorescence assay (IFA)

Parasites were allowed to invade HEK293T cells on coverslips at given MOIs and incubated for indicated time points. Next, cells were fixed with 4% formaldehyde for 20 min at room temperature. For parasite invasion assay, the extracellular parasites were stained with mouse anti-SAG1 antibody first and then washed three times using PBS. Then, cells were permeabilized and blocked by treatment with 0.1% Triton X-100 and 5% FBS. Subsequently, the coverslips were incubated with rabbit anti-ALD antibody and then with the respective secondary plus Hoechst (goat anti-rabbit Alexa Fluor 594 and goat anti-mouse Alexa Fluor 488; Invitrogen, USA). For detection of p65 nuclear translocation and expression of recombinant or truncated GRA15 proteins, coverslips of transfected cells were incubated with primary antibodies (mouse anti-FLAG and rabbit anti-p65; CST, USA) at indicated time points. Images were acquired using Olympus fluorescence microscopy system.

### Western blot

HEK293T cells in six-well culture plates at 60–80% confluency were transfected with an empty plasmid (as a control) or recombinant plasmids or left untreated for 30 h. TNF-α was used as positive control. Samples were washed with pre-chilled PBS, collected and lysed by RIPA lysis buffer (Beyotime, Beijing). Protein concentration was measured by BCA method according to the manufacturer’s instructions (Beyotime, Beijing). Then, cell lysates were boiled and subjected to SDS-PAGE and transferred onto polyvinylidene difluoride membranes (Millipore, USA). Membranes were blocked in TBST with 1% BSA for 2 h at room temperature and probed with primary antibodies (rabbit anti-p65, anti-phospho p65; Ser536; CST, USA) or mouse anti-IκB and anti-phospho IκB (Ser32/36; CST, USA). The mouse anti-GAPDH (EarthOx, USA) was used as a loading control. The primary antibodies were detected with HRP conjugated goat anti-mouse IgG or goat anti-rabbit IgG. The membrane blots were visualized and scanned by chemiluminescence kit (Bio-Rad, USA).

### Statistical analysis

All data analyses and graphs were performed using GraphPad Premier 8.0 software package (GraphPad Prism, San Diego, CA, USA). Statistical analyses were done from raw data with a one-way ANOVA and Student’s *t*-tests, where applicable. Data values were expressed as means ± standard deviation (SD). Differences between tested groups were considered significant if the *P* value was ≤ 0.05, indicated in the figures by asterisks (^*^P < 0.05, ^**^P < 0.01, ^***^P < 0.001 or ^****^P < 0.0001; NS, not significant). All experiments were repeated a minimum of three separate times.

## Results

### *Toxoplasma gondii* strains differ in activation of the NF-κB pathway

**Fig. 1:**
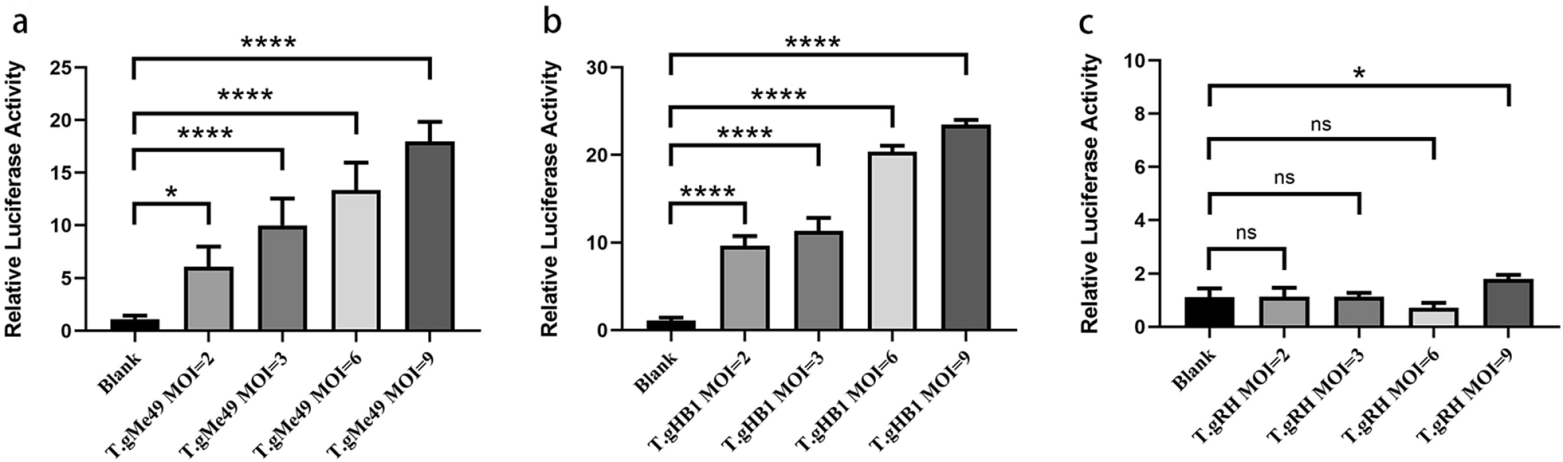
*Toxoplasma gondii* strains differ in their ability to activate the NF-κB promoter. **a** *Toxoplasma gondii* ME49s activate the NF-κB promoter. pNF-κB-Luc and pTL-TK plasmids were co-intransfected into HEK293T cells together with empty vector (blank) or infected with *T. gondii* ME49 at multiple MOIs. Eighteen hours post-infection, luciferase activity was measured. The relative luciferase activity was calculated by normalizing the fluorescence signals to the background. Error bars represent standard error. Data are presented as the average values of three independent experiments. **b** T.gHB1s activate the NF-κB promoter. **c** *Toxoplasma gondii* RHΔHX cannot activate the NF-κB promoter. (^*^P < 0.05; ^****^P < 0.0001; *NS* not significant)

**Fig. 2:**
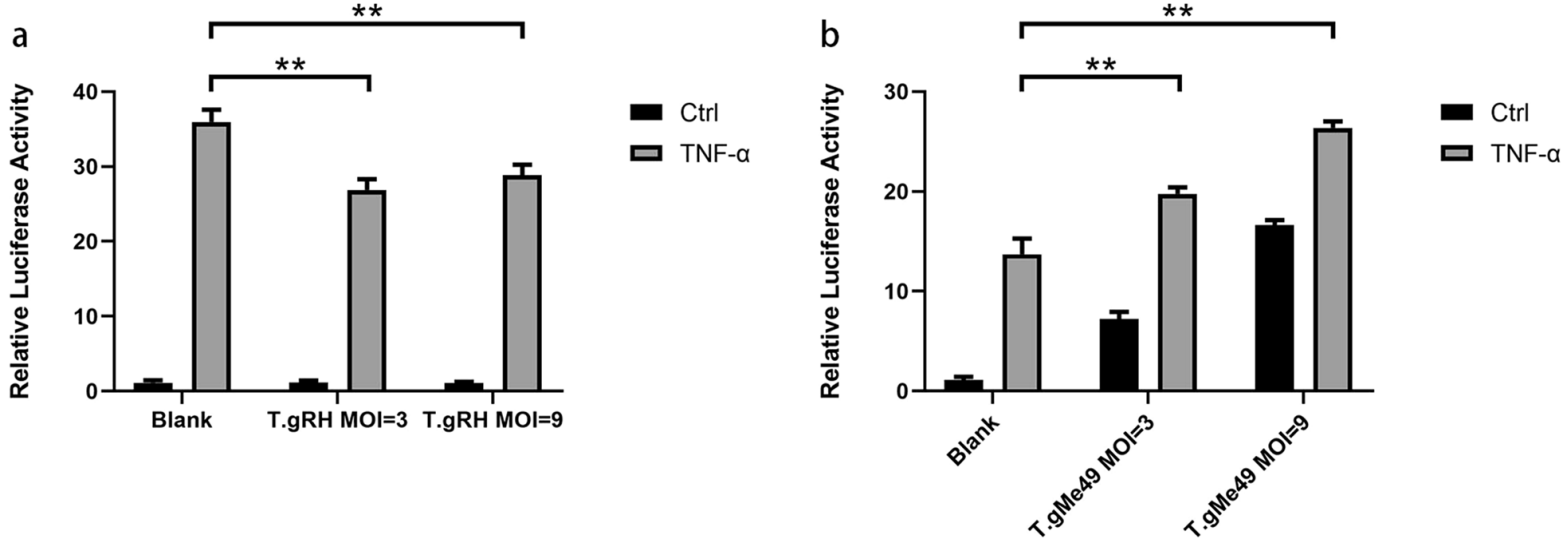
Different *T. gondii* strains at multiple MOIs inhibit activation of the NF-κB promoter. **a** pNF-κB-Luc and pTL-TK plasmids were co-intransfected into HEK293T cells followed by no treatment (blank) or infected with *T. gondii* RHΔHX. Six hours later, newly egressed tachyzoites were harvested, counted and added into the HEK293T cells as indicated. Eighteen hours post-infection, human recombinant TNF-α was added at a final concentration of 20 ng/ml and lasted for 6 h, and TNF-α was not added to the control (Ctrl). Twenty-four hours post-infection, cells were collected, lysed and luciferase activities were measured as recommended. The results of luciferase activity in each sample are presented as the average of three independent experiments. **b** *Toxoplasma gondii* strains from Me49 cannot inhibit activation of the NF-κB promoter. The results of luciferase activity in each sample are presented as the average of three independent experiments (^**^P < 0.01)

To detect the effects of different *Toxoplasma* strains on host NF-κB signaling, we infected HEK293T cells with T.gHB1 and the archetypal representative strains RHΔHX and ME49 at MOI of 2, 3, 6 and 9. A strong signal of NF-κB activation was observed after cell exposure to ME49 and T.gHB1. Increased luciferase activity was observed at higher MOIs (Fig. 1a and b), whereas no significant differences were detected between RHΔHX strain treatment and the blank control groups (Fig. 1c). To further investigate whether RHΔHX strain parasites have an inhibitory effect on NF-κB activation, HEK293T cells were infected with RHΔHX parasites at MOI of 3 and 9 or left untreated for 18 h and followed by treatment with TNF-α for 6 h. ME49 strain (a type 2 strain) was used as control in this experiment. Results showed that TNF-α alone was sufficient to induce a strong NF-κB response (Fig. 2a). However, cells that were pre-treated with RHΔHX parasites showed weakened reaction to TNF-α stimulation as reflected by downregulation of luciferase activity (*P* < 0.01) (Fig. 2a), which suggests that RHΔHX parasites exert an inhibitory effect on NF-κB signaling in HEK293T cells. In contrast, ME49 parasites showed a synergistic effect with TNF-α on host cell NF-κB signaling (Fig. 2b). Taken together, these results demonstrate that different strains of *Toxoplasma* differ in their ability to activate the host NF-κB signaling pathway. Unlike type 1 RHΔHX strain, T.gHB1 strain imposes a positive effect on NF-κB signaling in HEK293T cells.

### Parasite invasion ability does not contribute to differences in NF-κB activation

**Fig. 3:**
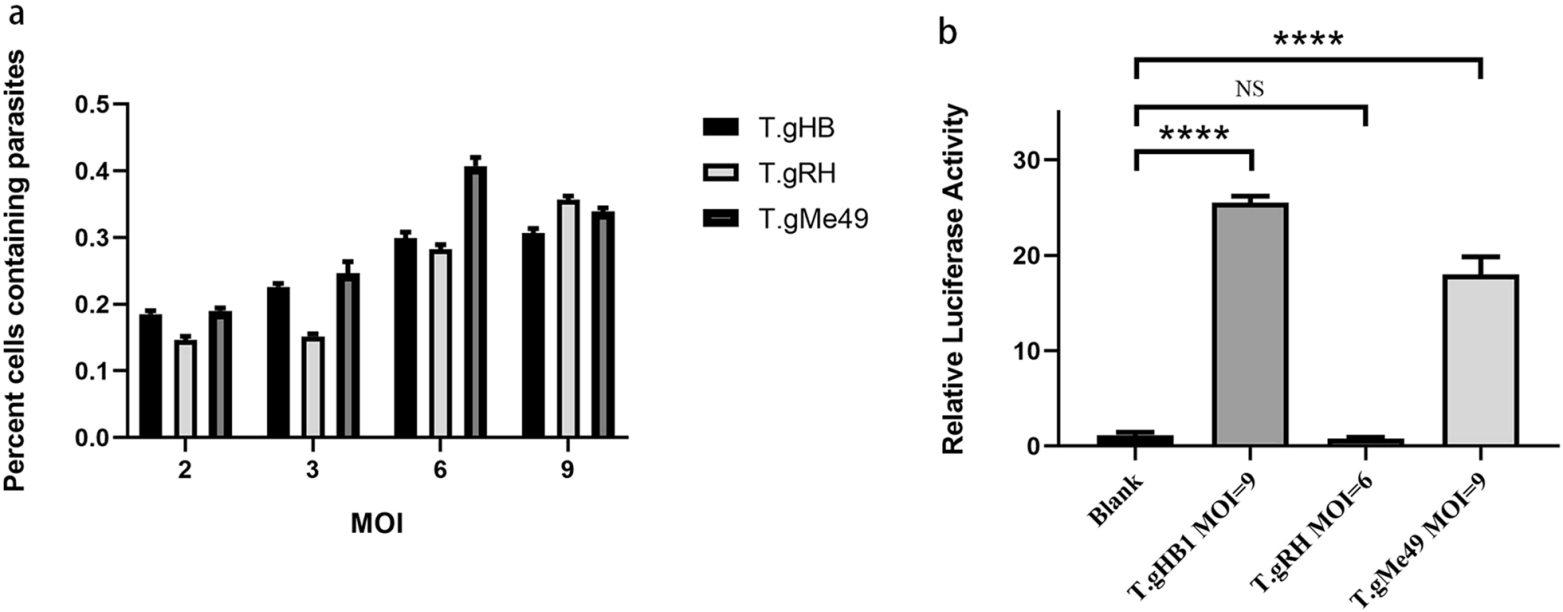
Differences in *T. gondii* parasite invasion ability do not affect the strain-specific activation of NF-κB promoter. HEK 293 T cells were infected with *T. gondii* for 18 h and fixed; the extracellular and intracellular parasites were distinguished by differential IFA staining. **a** The overall parasite invasion rate for each strain in HEK293T cells. The percentage of infection was determined by counting at least 15 HEK293T cells for each strain at each MOI. **b** MOI for different parasites was adjusted to make comparative parasite invasion rate in HEK293T cells; 18 h post infection, the luciferase activity was measured in cell lysates. Data are presented as the mean relative luciferase intensity with standard deviation of three independent experiments (^****^P < 0.0001 and *NS* not significant)

The invasion ability of *Toxoplasma* may be different for different strains and thus may lead to significant changes of host cell responses to parasite infection. To exclude the possibility that there are differences in NF-κB responses in HEK293T cells to different *Toxoplasma* parasites, we conducted parasite invasion assays of different parasite strains in HEK293T cells at multiple MOIs of 2, 3, 6 and 9. Results indicated that different parasite invasion rates were observed at the same MOI between different parasite strains (Fig. 3a). To achieve comparable parasite invasion rates in HEK293T cells, the MOIs of six for RHΔHX and nine for T.gHB1 and ME49 were selected in all subsequent assays. The results revealed that T.gHB1 strain as well as ME49 showed an obvious activation level of the NF-κB pathway as previously described (Fig. 3b). However, no detectable NF-κB signal was detected in the RHΔHX infected cells (Fig. 3b). These data suggest that the differences in activation of host NF-κB pathway between *Toxoplasma* strains were not determined by parasite invasion ability in HEK293T cells.

### GRA15 of T.gHB1 is able to activate the NF-κB pathway

**Fig. 4:**
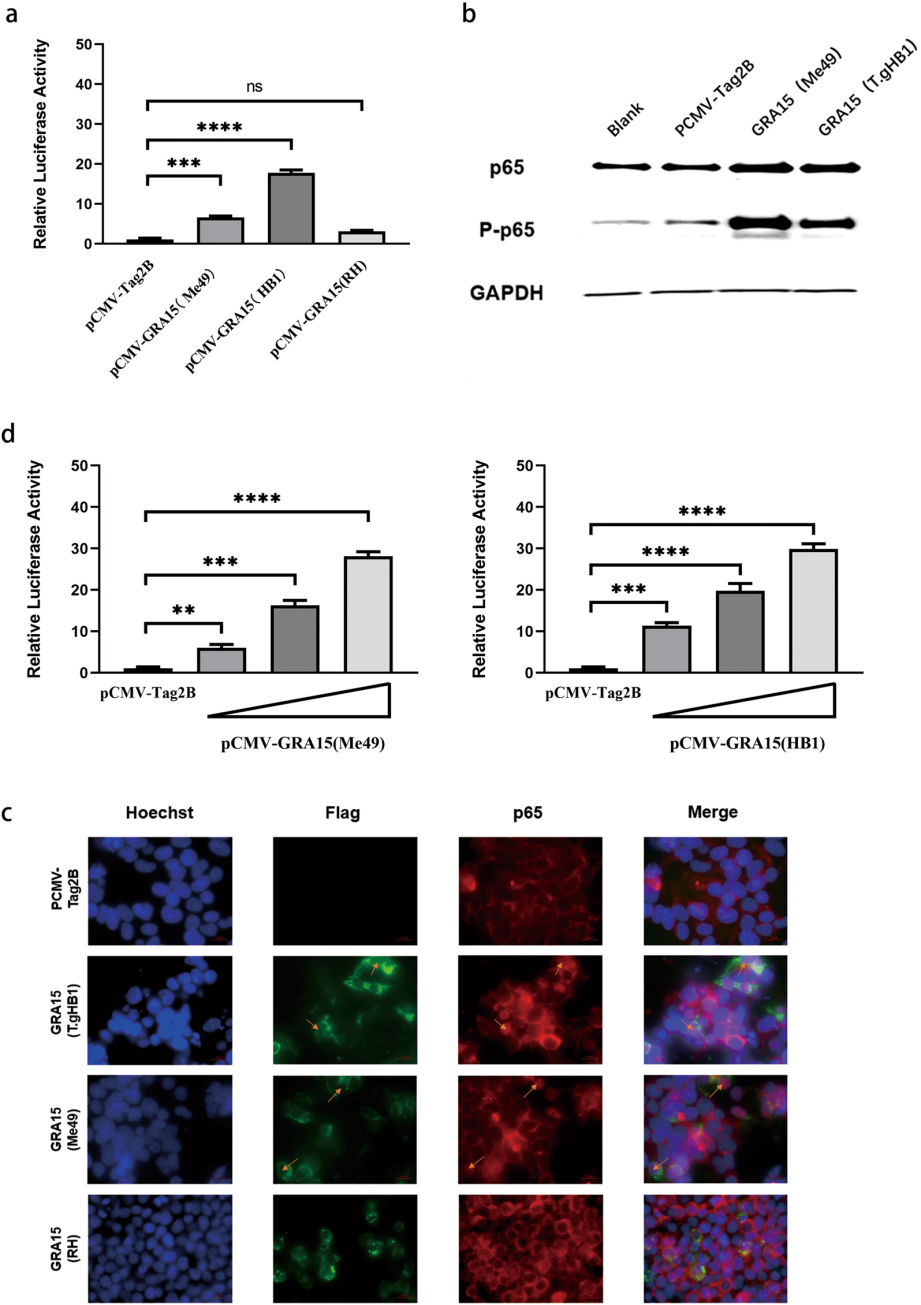
T.gHB1 GRA15 activates NF-κB promoter. **a** T.gHB1 GRA15 expression plasmid was co-transformed with NF-κB-Luc and pTL-TK plasmids into HEK293T cells. After 24 h, the luciferase activity was measured in cell lysates. Data are presented as the values with standard error of three independent experiments. **b** HEK293T cells were co-transfected with different GRA15 encoding plasmids for 30 h. The expression levels of P65 and phosphor-P65 were detected by Western blotting. **c** Fluorescence microscopy of p65 in 293 T cells transfected with empty vector (PCMV) or vector for FLAG-tagged GRA15. Hoechst, DNA-intercalating dye. Original magnification. Nuclear translocation of p65 was detected by IFA. **d** GRA15 expression plasmid was co-transformed with NF-κB-Luc and pTL-TK plasmids into HEK293T cells with increasing amounts of GRA15 plasmid. Data are presented as the mean relative luciferase intensity with standard deviation of three independent experiments (^**^P < 0.01; ^***^P < 0.001; ^****^P < 0.0001; *NS* not significant)

A previous report has shown that the *Toxoplasma* effector protein GRA15, a novel dense granule protein, from type 2 parasites can contribute to host NF-κB activation, whereas a similar protein of the type 1 parasite (e.g., RHΔHX) cannot [24]. Therefore, we set out to examine whether the GRA15 protein of the recently discovered T.gHB1 strain can exert an effect on NF-κB pathway in HEK293T cells. To do so, the plasmids encoding T.gHB1 GRA15, RHΔHX GRA15, ME49 GRA15 as well as an empty plasmid (as a control) were individually transfected into HEK293T cells along with a luciferase reporter plasmid and an internal control plasmid as described in the Materials and Methods section. Luciferase activities were detected, and the results suggested that expression of T.gHB1 GRA15 induced high levels of NF-κB activation (Fig. 4a, d) and demonstrated that the activation was dose-dependent, whereas expression of RHΔHX GRA15 barely induced NF-κB activation. These data suggest that the GRA15 proteins of type 2 strain and T.gHB1 can mediate NF-κB activation, whereas the RHΔHX GRA15 cannot.

NF-κB activation involves a complex cascade of host cell signaling that includes phosphorylation and nuclear translocation of the transcription factors [26]. To investigate the effects of T.gHB1 GRA15 on NF-κB transcription factor function, HEK293T cells were cultured in six-well culture plates and transfected with plasmids encoding T.gHB1 GRA15 or the ME49-derived GRA15 as a positive control. The total protein expression profiles of p65 and phosphorylated p65 (p-p65) were determined by Western blotting. The results showed a significant increase of p65 and phosphorylated p65 levels in cells that express T.gHB1 GRA15 and ME49 GRA15 compared to the control cells (Fig. 4b). In addition, translocation of the p65 protein into the cell nucleus was also verified by co-staining with nuclear Hoechst (Fig. 4c). These results suggest that T.gHB1 GRA15 alone can activate NF-κB pathway, which results in p65 phosphorylation and nuclear translocation, while RHΔHX GRA15 cannot.

### Identification of functional domain of T.gHB1 GRA15 that can activate the NF-κB promoter

**Fig. 5:**
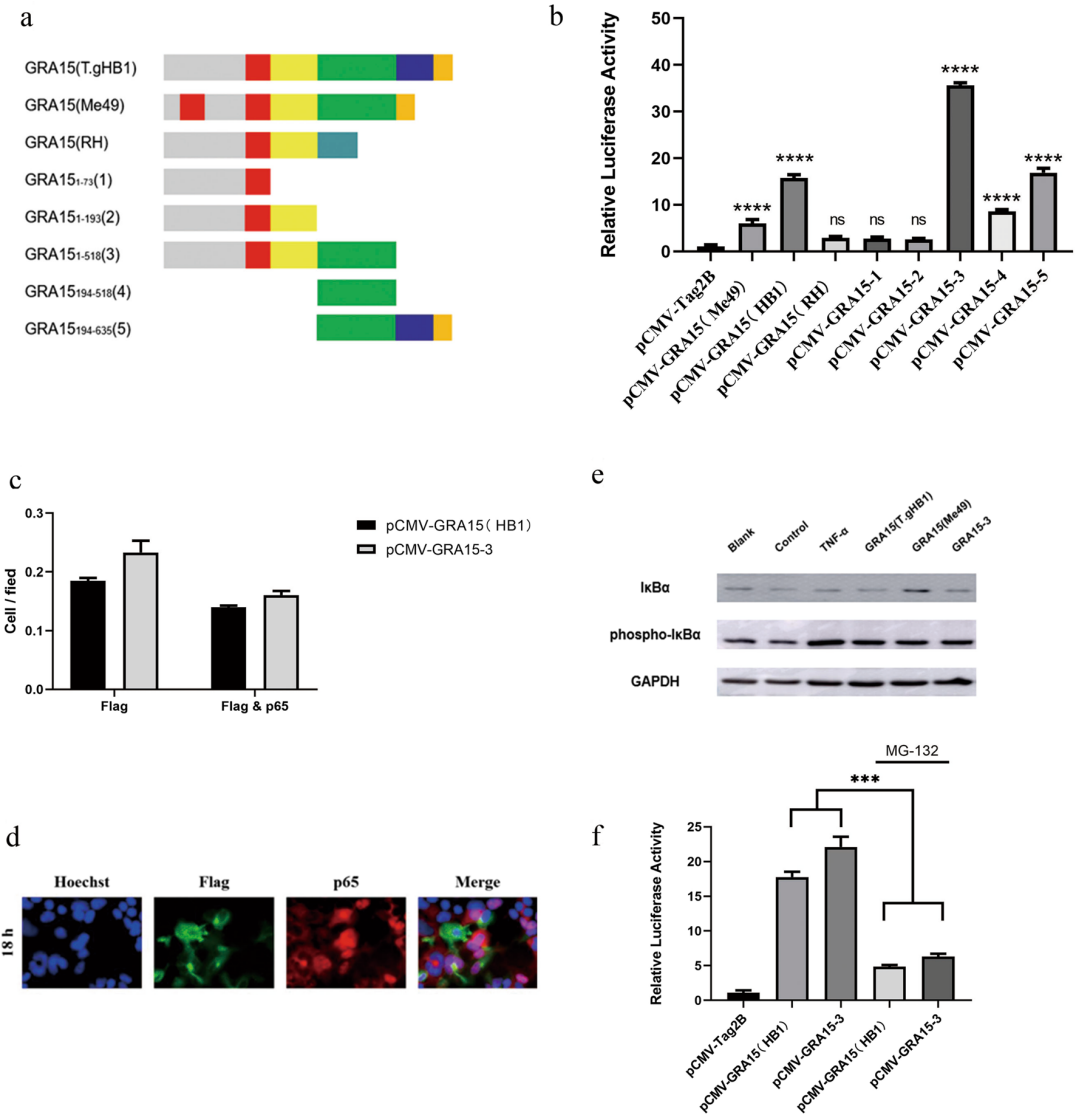
Identification of potential functional domains in T.gHB1 GRA15 for NF-κB promoter activation. **a** Illustration of full-length and truncated GRA15 proteins. Bioinformatics analysis was performed on the full length of GRA15 (T.gMe49), GRA15 (T.gHB1) and GRA15 (T.gRH). GRA15 (T.gHB1) and GRA15 (T.gRH) have a transmembrane region (the area marked in red). GRA15 (T.gMe49) has two transmembrane regions (areas highlighted in red) and lacks 518–602 AA regions compared to GRA15 (T.GHB1). Five truncated plasmids were constructed according to this design. **b** HEK293T cells were co-transfected with NF-κB luciferase reporter plasmid along with each of the GRA15-containing plasmids, and after 24 h, the luciferase activity was measured in cell lysates. Data are presented as the values with standard error of three independent experiments. **c** The FLAG-tagged GRA15-containing plasmid GRA15_1-518_ (3) was transfected into HEK293T cells for 24 h, and p65 was stained by IFA. Hoechst, DNA-intercalating dye. **d** The percentage of cells expressing FLAG tag was calculated, and p65 positivity in the cell nucleus was assessed by co-staining with the FLAG-positive cells. Data were generated in three independent experiments (mean ± SD; *n* = 3). **e** HEK293T cells were transfected with different GRA15-containing plasmids as indicated for 30 h. TNF-α was used as positive control. Expression levels of IκBα and of phosphorylated IκBα were detected by Western blotting. GAPDH was used as a loading control. **f** HEK293T cells were co-transfected with NF-κB and pTL-TK luciferase reporter plasmid along with each of the GRA15-containing plasmids, and 18 h post, MG-132 was added and lasted for 6 h. After 24 h the luciferase activity was measured in cell lysates. Data are presented as the values with standard error of three independent experiments (^***^P < 0.001; ^****^P < 0.0001; *NS* not significant)

Bioinformatics analysis revealed a distinct protein structural pattern between T.gHB1 GRA15 and ME49 GRA15 (Fig. 5a). As compared to type 2 GRA15, GRA15 from T.gHB1 contains only one transmembrane region and a longer C terminal extension (518–602 aa). To further identify the potential functional domains required for NF-κB promoter activation, we constructed plasmids to express GRA15 truncated protein-encoding sequences and introduced each of them into host cells (Fig. 5a). Our assays indicated that expression of the truncated proteins that include GRA15_1-518_, GRA15_194-518_ and GRA15_194-635_ mediated relatively high levels of NF-κB driven transcription of luciferase gene expression (*P* < 0.01) (Fig. 5b). GRA15_1-193_ showed no effects on NF-κB promoter activation. When cells were transfected with GRA15_1-518_ plasmids, the resulting protein induced the strongest fluorescence signals in HEK293T cells (Fig. 5b). As expected, the GRA15_1-518_ induced about an equal level of nuclear translocated p65 compared to the full-length T.gHB1 GRA15 protein (Fig. 5c). In addition, the effect of GRA15_1-518_ on p65 nuclear translocation was confirmed by IFA (Fig. 5d). In summary, these data demonstrate that 1–518 aa of T.gHB1 GRA15 was mainly responsible for efficient NF-κB activation in HEK293T cells.

NF-κB subunit activation and nuclear translocation relied greatly on the phosphorylation and degradation of NF-κB inhibitor, IκBα, by proteasome [27]. We therefore determined the overall expression levels of IκBα and phosphorylated IκBα (P-IκBα) by immunoblotting. Results revealed that the amounts of phosphorylated IκBα in cells transfected with ME49 GRA15, T.gHB1 GRA15 and T.gHB1 GRA15_1-518_ plasmids were significantly higher (*P* < 0.05) than in the controls. These results indicate that T.gHB1 GRA15 can activate the classic NF-κB pathway via the phosphorylation and inactivation of the IκBα protein (Fig. 5e). Furthermore, we inhibited the action of the proteasome and showed that the activation of GRA15 was inhibited (Fig. 5f).

## Discussion

*Toxoplasma gondii* is an important member of Apicomplexan protozoan, which infects many animals and humans worldwide [28]. Previous studies have reported that *Toxoplasma* strains or isolates display marked genetic diversity with geographic distribution, thus resulting in differences in disease pathogenesis and host signaling modulations by the different parasites [29]. In the present study, we describe the ability of a type 1 *Toxoplasma* isolate that was isolated in Central China to exhibit a distinct characteristic in host signaling modulation compared to other known classic type 1 parasites. This newly discovered *Toxoplasma* strain named T.gHB1 has previously been reported by us to exhibit an alternative virulence determinant compared to other typical parasites of the type 1 strain, such as the RHΔHX strain [19].

In the current work, we presented experimental evidence for another polymorphic aspect of T.gHB1 in host cellular NF-κB signaling. Infection of HEK293T cells by T.gHB1 significantly increased the NF-κB-dependent transcription of luciferase reporter gene, similar to the type 2 ME49 strain (Fig. 1b). Furthermore, a stronger luciferase activity was correlated with increased parasite burdens by the T.gHB1 and ME49, but not by a typical type 1 strain RHΔHX (Fig. 1). In contrast, we observed an inhibitory effect of RHΔHX parasites on NF-κB activation as illustrated by a reduction of luciferase activity in TNF-α primed cells (Fig. 2). To exclude the possibility that the divergence in NF-κB modulation was caused by parasite invasion ability in HEK293T cells, cellular parasite invasion rates were tested at multiple MOIs and were accordingly adjusted in all subsequent assays. Taken altogether, our data suggest that the differences of NF-κB activation in HEK293T cells by different strains of the parasite was not due to parasite invasion ability, but it is the genetic differences of the parasites that may explain their ability to mediate NF-κB activation in HEK293T cells.

We observed that HB1 and Me49 strains significantly activated the NF-κB pathway in 293 T cells. Interestingly, RH strains had a certain inhibitory effect. It is proved that different strains of the worm have completely different regulation of the host signaling pathway. Different from GRA15, ROP18 has been shown to inhibit the NF-κB pathway in the host [30], demonstrating that *T. gondii* can use different effector proteins to regulate different responses to the same signaling pathway in the host. This may account for the wide range of *T. gondii* hosts.

Previous studies have reported that host NF-κB pathway can be regulated by GRA15 from type 2 *Toxoplasma* parasites [24]. We therefore set out to test the potential impact of T.gHB1 GRA15 on the modulation of NF-κB cascade in HEK293T cells. Our results indicate that T.gHB1 GRA15 has a different topological structure compared to type 2 GRA15. T.gHB1 GRA15 contains 635 aa residues consisting of a predicted transmembrane with a long C terminal extension. The impact of T.gHB1 GRA15 on NF-κB pathway was valued by assays. Our dual luciferase results showed that the expression of T.gHB1 GRA15 in HEK293T cells can remarkably upregulate the levels of fluorescence signals compared to the control. Furthermore, our Western blotting results combined with p65 co-staining with Hoechst nuclear dye confirm the ability of the T.gHB1 GRA15 to mediate phosphorylation and nuclear translocation of the p65 protein to modulate the NF-κB pathway.

GRA7 and GRA14 were reported, which can partially activate the NF-κB signaling pathway, whereas GRA15 was essential for NF-κB activation. Cells infected with the PruΔgra15 parasite showed reduced phosphorylation of inhibitor-κBα [31]. It shows that GRA15 is an important factor activation of host NF-κB signaling pathway. Furthermore, GRA15 was found to interact with TNF receptor-associated factors (TRAFs) to activate this pathway. TRAFs are the upstream functional junction protein of NF-κB transcription factor. The loss of TRAF binding sites in GRA15 significantly reduces its NF-κB pathway activation, while TRAF2 knockout cells also impair GRA15-mediated NF-κB activation [32].

As dense granule proteins (e.g., GRA15) are generally structurally polymorphic and possess little homology to each other [33, 34], and the lack annotation of the GRA15 functional domains, in particular, impedes our understanding of the precise mechanism by which GRA15 activates host NF-κB pathway. We therefore set out to map the functional domains of the T.gHB1 GRA15 by characterizing several truncated versions of this protein in HEK293T cells to mediate NF-κB activation. Our data suggest that 194–518 aa of T.gHB1 GRA15 are required for optimal activation of the NF-κB promoter. Although the underlying mechanism of how T.gHB1 GRA15 activates the NF-κB promoter is unknown, we predict that 1–193 aa of the T.gHB1 GRA15 may have a coenzyme like role or act as a scaffold for the full-length T.gHB1 GRA15 protein.

IκBα is an inhibitor of the NF-κB subunits in cells at the resting state. Once cells are activated by stimulus treatment or pathogen infection, IκBα can be phosphorylated and degraded by proteasome, thus allowing the NF-κB subunits to be phosphorylated and transported into the cell nucleus [27]. Our results indicate that the full-length T.gHB1 GRA15 and its GRA15_1-518_ version can significantly increase the amount of phosphorylated IκBα (Fig. 5e), which suggests that T.gHB1 GRA15 initiates NF-κB activation by phosphorylation of IκBα, similar to what has been observed with the ME49 GRA15.

## Conclusion

We provide experimental data to show that a newly discovered *Toxoplasma* isolate T.gHB1 from Central China that belongs to type 1 parasites is unique in that, unlike the classical type 1 strains, T.gHB1 can exert a positive effect on the host NF-κB pathway. The molecular mechanism and biological significance of T.gHB1 on host NF-κB manipulation deserve more attention in future studies.

## Data Availability

Data supporting the conclusions of this article are included within the article.
